# Various Types of Virtual Reality-Based Therapy for Eating Disorders: A Systematic Review

**DOI:** 10.3390/jcm11174956

**Published:** 2022-08-24

**Authors:** Julia Ciążyńska, Janusz Maciaszek

**Affiliations:** Department of Physical Activity and Health Promotion Science, Poznan University of Physical Education, 61-871 Poznan, Poland

**Keywords:** health, virtual reality, head-mounted display, eating disorders, therapy intervention, binge eating disorder, anorexia nervosa, bulimia nervosa, body image disturbance

## Abstract

(1) Background: Interactive VR (virtual reality) environments (i.e., using three-dimensional graphics presented with a head-mounted display) have recently become a popular professional tool for the treatment of patients with eating disorders (EDs). However, there are no published review reports that have analyzed the original papers between 2015 and 2021, which additionally focused only on HMD (head-mounted display) 3DVR (three-dimensional virtual reality) exposure and included only three therapeutic categories for ED patients. (2) Methods: The EbscoHost and Scopus databases were searched to identify relevant papers on VR research employing VR in the assessment and treatment of eating disorders. (3) Results: In addition to the known therapeutic divisions for ED, there are new forms of therapy based on 360 cameras, eye-tracking, and remote therapy. (4) Conclusions: The potential of VR in combination with different therapies may offer an alternative for future research. More rigorous testing, especially in terms of larger sample sizes, the inclusion of control groups or multisessions, and follow-up measures, is still needed. The current state of research highlights the importance of the nature and content of VR interventions for ED patients. Future research should look to incorporate more home-based and remote forms of VR tools.

## 1. Introduction

Virtual reality (VR) is a modern technology that can greatly enhance existing therapies with the possibility of enhancing treatment outcomes. Through the creation of immersive computerized 3D experiences, patients interact normally with stimuli representing real-life scenarios while simultaneously benefiting from a medical, supervised environment [1]. Interactive VR environments (i.e., using three-dimensional graphics presented with a head-mounted display) have recently become a popular professional tool for the treatment of patients with EDs. VR is entering the field of medicine, and it seems that the role of this tool will grow rapidly.

Patients with EDs are characterized by pathological eating habits, the pursuit of ideal body shapes, and a tendency to overestimate their weight, according to DSM 3,4,5 [1]. AN (anorexia nervosa), BN (bulimia nervosa), and BID (body image disturbance) problems are now among the most severe mental disorders in adolescents and young adults. Eating disorders are connected with serious psychological and medical effects [2] and a high risk of death [3]. While therapies for EDs show reasonable efficacy, on average, only 50% of those therapies make full progress, with full progress being a full recovery [4]. Innovations are still needed to improve treatment results.

Therapies and interventions are strategies of care, but their main difference is scope. Interventions are usually designed to motivate someone to take a certain action, such as seeing themselves in a better light or just starting treatment. Treatment has more complex goals, such as changing habits or attitudes. Many researchers have seen a great potential for VR-based exposure interventions in the treatment of eating disorders [5,6,7,8]. There are advantages to VR-based therapy and VR body exposure in the context of several psychopathologies, especially mental disorders. Some reviews suggest that VR-based interventions are at least as effective as those based on CBT (cognitive behavioral therapy) programs, which is the first level of treatment before CET (cue-exposure therapy) sessions, VR-CET (cue-exposure therapy in virtual reality), or A-CET (analog cue-exposure therapy). However, it should be noted that VR-based therapies often mix multiple components in their protocols. Of the many applications of VR in the treatment of EDs, VR-CET has empirical support at present [5,9,10,11]. Additionally, CET has proven its efficacy in treating patients with bulimia nervosa and binge eating disorder who are resistant to standard treatment [12]. The VR market is still developing, and the possibilities of its use are increasing, which is why new alternative types of previously known ED therapies are appearing. In this paper, we review and summarize the findings from the literature on the adoption of VR-based exposure therapies such as CET, body exposures, and alternative VR therapies for the assessment or treatment of EDs. However, there are no published review reports that have analyzed the original papers between 2015 and 2021, which additionally focused only on HMD 3DVR exposure and included only three therapeutic categories for ED patients. Knowing that there are already published ED and VR reviews [1,13,14,15], we wanted ours to cover additional issues and thus stand out from the rest. This paper is centered around two main research questions: what VR-based therapies exist; what are the potential future trends for VR-based therapy in eating disorders?

The review will focus on the human population, namely, patients of all ages, gender, and ethnicity diagnosed with EDs: AN, BN or BED, or OSFED, according to the Diagnostic and Statistical Manual of Mental Disorders (DSM 3, 4, or 5). All studies that meet the inclusion criteria and their respective interventions and comparison conditions are reviewed. Intervention studies were included if the study’s purpose was VR exposure of the assigned ED. Intervention studies were not included if the paper was not based on a virtual-reality-based therapy but only augmented reality therapy.

This paper includes 15 articles published in 2015 and after, and thus, it expands on previous findings by presenting a wider and updated overview of the potential of virtual-reality-based therapy.

## 2. Materials and Methods

### 2.1. Search Strategies

We chose papers from 2015 to 2021 to review because other reviews worked on papers from 1998 to 2015 and early 2016 [1,13,14,15]. After deliberation with the two independent researchers, EbscoHost as a bibliographic database and Scopus as an electronic database were identified to be searched. The search was based on the following index terms: ‘virtual reality’ [all fields] AND (‘treatment OR therapy OR program OR intervention’ [all fields] AND ‘eating disorder’ [all fields] NOT ‘review OR meta-analysis’ [all fields]. The review was registered on the PROSPERO webpage, which is an international register of systematic reviews. The registration number is CRD42021272378. We used PRISMA (Preferred Reporting Items for Systematic Review) [16] to identify and analyze the scientific literature on VR research in EDs. The PRISMA checklist is available in Supplementary S1.

### 2.2. Study Eligibility

Articles that mentioned an interactive VR environment (i.e., using three-dimensional graphics presented with a head-mounted display), conducted VR exposure of the assigned EDs, were published in a peer-reviewed journal and were open-access, were conducted with human subjects, and were published between 2015 and October 2021 were included in our review. Articles that were not written in English or Polish, were not related to HMD 3DVR but to augmented reality only, or were a meta-analysis, dissertation, protocol article, opinion letter, conference article, or a review were excluded from our review.

### 2.3. Data Extraction 

Data were extracted with the help of a standardized Excel form. The extracted data included: the first author and year of publication (reference), study objectives, study design, diagnostics, sample sizes, control groups, sessions, methods, interventions, and outcomes.

### 2.4. Quality Analysis of Studies

**Figure 1:**
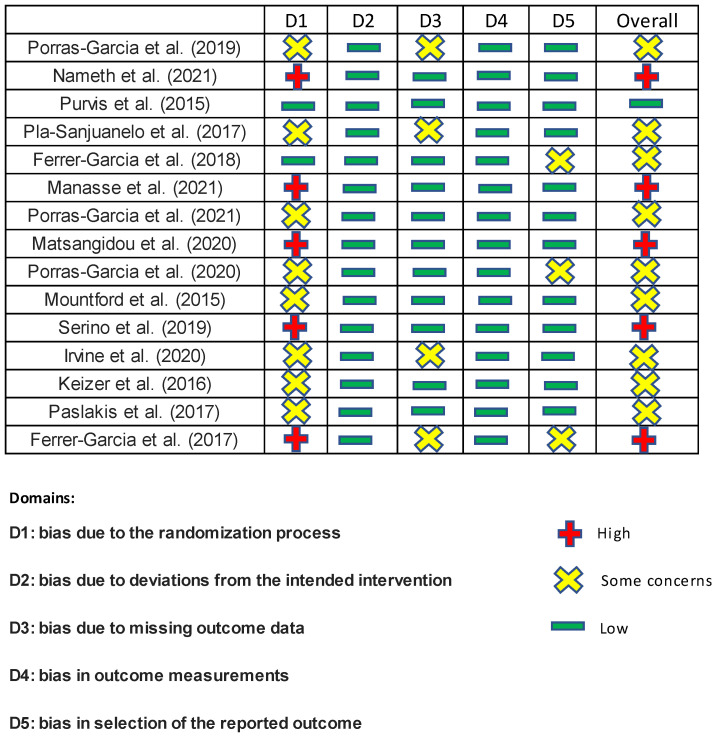
Risk of bias of all included papers [5,6,7,8,9,10,11,18,19,20,21,22,23,24,25].

The risk of bias was independently assessed by two researchers using the latest version of the Cochrane Collaboration Risk-of-Bias tool (RoB 2.0 data) [17]. The tool includes algorithms that give a proposed risk of bias score for each domain at three levels: high, some concern, and low. Low risk of bias means that the study is judged to be at low risk of bias for all domains for this result. Some concern means that the study is judged to be of some concern in at least one domain for this result. High risk of bias means that the study is judged to be at high risk of bias in at least one domain for this result or the study is judged to be of some concern in multiple domains in a way that substantially lowers confidence in the result. All studies were assessed in five domains: bias due to the randomization process, bias due to deviations from the intended intervention, bias due to missing outcome data, bias in outcome measurements, and bias in the selection of the reported outcome. As a result, five studies were rated as having a high risk of error, nine as having an unclear risk of error, and one as being low risk. The details are shown in Figure 1.

## 3. Results

### 3.1. Description of Studies

**Figure 2:**
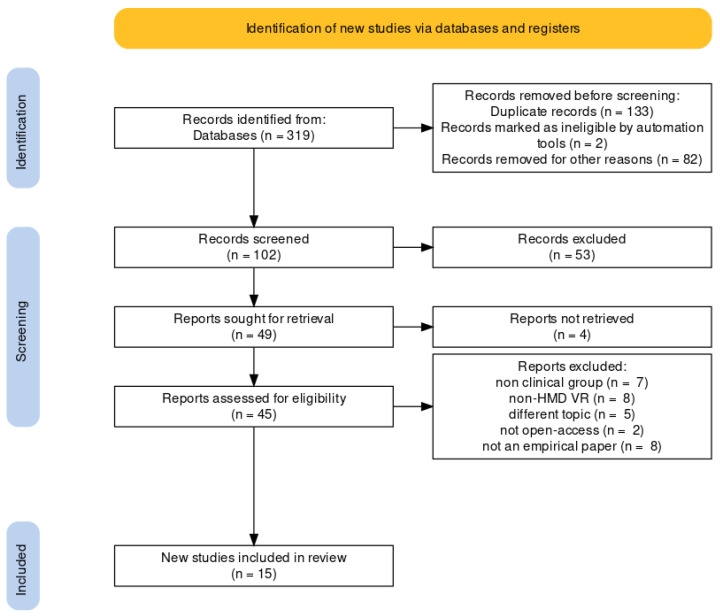
Overview of the screening and paper selection process (PRISMA flowchart).

Despite the search by keywords and the removal of duplicates by the system, there were articles that did not meet the criteria for inclusion in the research. The search was completed in October 2021, resulting in a total of 180 papers from EbscoHost and 139 papers from Scopus. After removing duplicates between the databases and between the articles themselves and also removing records that were ineligible, articles published before 2015, and articles published in any language other than English or Polish, 102 papers remained. The selection was completed using exclusion based on title, abstract, and the entirety of the paper. After selections based on the title, 45 papers remained. The last section, based on the abstract and the entirety of the paper, gave us the goal of 15 papers. We also manually searched for a research group. We did not take into account papers that were not focused on virtual-reality-based therapy using 3D in an interactive VR environment (i.e., using three-dimensional graphics presented with a head-mounted display), n = 8; were not empirical papers but review articles, meta-analyses, dissertations, protocol articles, opinion letters, or conference articles, n = 8; were about disorders other than eating disorders (anorexia nervosa or bulimia nervosa or disordered eating or binge eating), n = 5; were not accessible online, n = 2; contained healthy participants without eating disorders, n = 7. Figure 2 shows a flowchart overview of the identification. See Supplementary S2 [5,6,7,8,9,10,11,18,19,20,21,22,23,24,25] for a list of the selected papers.

**Figure 3:**
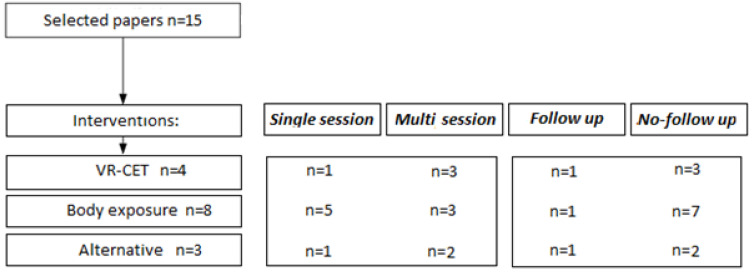
Overview of the included papers.

A few general observations were made using the data set of papers. Of the 15 selected papers, all of them are intervention papers: VR-CET, body exposure, and alternatives. In addition, 53% (8/15) of the intervention papers described a multisession intervention, stretching from 2 to 12 weekly sessions. Furthermore, 37.5% (3/8) of these multisession interventions reported at least one follow-up measure, ranging from 2 weeks to 12 months after the final session. Follow-up measurements are a great asset to intervention studies as initial effects might fade over time. Figure 3 shows a flowchart indicating the division of the papers.

### 3.2. What VR-Based Therapies Exist?

Different VR-based therapies for EDs were found. Body exposure using VR therapy was the therapy most frequently reported (n = 8). The next most frequently reported therapy (n = 4) was CET in VR (VR-CET). In addition, VR has been combined with different interventions, such as MUVR-exposure, VR-ICT, or CET with VR and 360 cameras, which are not the standard form of the well-known interventions listed above (n = 3). Each subgroup (VR body exposure, VR-CET, and alternative forms) presents an intervention for a specific ED variety. Table 1, as an abbreviated version of Supplementary S2, shows all articles included in this systematic review, including references, diagnoses of patients/participants, and methods (for questionnaires, protocols, scales, measures, hardware, and software).

#### 3.2.1. VR-Exposure Therapy

A total of eight papers on VR body exposure therapy or a variation were included in the review. Half of them [7,18,22,24], which are described in this paragraph, were based on BD and BID populations. The papers proved that VR body exposure therapy has been successfully used as a satisfactory tool for body image assessment and represents an innovative method to specifically reduce bodily concerns. Three studies in the selected VR body exposure therapy papers had only one single session of treatment [7,22,24]. Only Porras-Garcia et al. [22] did not have a control group for research; the rest of them did [7,18,24]. Additionally, the Porras-Garcia et al. [22] paper had a mix of female and male populations; the rest of the papers were based on the female population.

The other half of the papers [8,19,23,25] regarding VR body exposure therapy were specialized in the treatment of AN and BD. Porras-Garcia et al. and Keizer et al. [19,23] focused on one-off sessions based on FBI (full body illusions). However, the two research papers had similar study objectives that relied on emotions. Keizer et al. [19] wanted to prove that a VR FBI affects the body size estimation of body parts in a more emotionally salient way than an FBI by hand, and Porras et al. [23] wanted to evaluate the usefulness of VR body exposure software for the assessment of important body-related cognitive and emotional responses in AN. Furthermore, both hypotheses proved correct. 

The remaining papers [8,24] focused on multisessions and also had similar study objectives, such as reporting the use of the VR protocol within a multidisciplinary treatment of AN. However, they differed in terms of the methods and research groups. Serino et al. [24] focused only on one patient with AN who underwent an intensive, multidisciplinary outpatient treatment, namely, three sessions of a VR-based body-swapping illusion. Porras-Garcia et al. [8] had experimental and control groups for both AN patients. The experimental group received the TAU (treatment as usual) treatment and five additional sessions of body exposure therapy, while the control group received only TAU. ED symptoms were clearly reduced in both groups of research.

#### 3.2.2. VR-CET Intervention

A total of four papers on VR-CET or a variation were included in the review. VR-CET has been successful in reducing binge and purge episodes [5,9,10] over repeated exposures in most experiments [5,9,10,11]. One study in the selected VR-CET papers had only a single session of treatment [11], but their sample size was the largest at n = 193; it was also the only study that had a control group (n = 135). All studies on the VR-CET group were based on BN and BED patients. Additionally, one study by Ferrer-Garcia et al. [9] presented a follow-up analysis on the effectiveness of VR-CET on BN and BED patients and focused on the evolution of symptoms assessed after 6 months. The rest of the papers focused on the research period without a follow-up section.

Each article was based on the VR-CET intervention in which the patient selected and rated a certain number of craved food products that caused an increase in anxiety and craving levels [5,9,10,11]. The number of products fluctuated from 10 [11] to 30 [5,9,10], and exposure time also fluctuated from 20 [5] to 45 [10] seconds per image of food. The areas of the exposures were the same in each research paper: dining room, kitchen, bedroom, and restaurant/diner/cafeteria [5,9,10,11], where the patients usually binge in real life.

Three papers described the implementation of VR-CET after other interventions. First, Ferrer-Garcia et al. [9] implemented VR-CET after a CBT program, which is the first level of treatment before VR-CET or A-CET sessions, thereby recruiting for research a population that was still characterized by active episodes of binge eating. This study, the only one using this comparison, randomly divided the two groups into different treatments. One group attended the VR-CET sessions, and the other attended the A-CET sessions (additional cognitive behavioral therapy). The obtained reductions were greater after VR-CET versus A-CET regarding binge and purging episodes as well as a decrease in self-reported tendencies to engage in overeating episodes. The other two papers [5,10] implemented a VR-CET intervention after a CBT session. Ferrer-Garcia et al. [5] proved that second-level treatment is effective for patients who fail the first-level treatment. Nameth et al. [10] found that the integration of VR (both immersive and semi-immersive) into cue-exposure therapy for BED, like BN, enhances treatment outcomes for refractory patients compared to CBT alone.

#### 3.2.3. Alternative VR Exposure

A total of three HMD 3DVR ED interventions that relied on an alternative component were identified: one VR adaptation of the MUVR (multi-user virtual reality system) intervention, one paper on jogging training, and one on gamified VR-ICT (inhibitory control training). Two studies on VR alternative therapy groups were based on BN and AN female patients and had only one session of treatment [6,21]. One study by Manasse et al. [20] recruited participants with once-weekly LOC (loss of control) eating; mainly women participated in the research (71.4%). What distinguishes the Manasse et al. [20] paper is that they had multisession home training and a follow-up period. It is worth noting that in the case of the Matsangidou et al. [6] article, the training did not take place at home; the patients and trainers never met during their sessions because the sessions were held in the same building but in different rooms, 50 m apart. Hence, referring to these two articles [6,20], the training took place remotely. The positive effect of blind psychotherapy (i.e., remote technology-based therapy in which the patient and the therapist are not in the same physical space) has been documented by studies that have examined blind telephone counseling. These studies report that telephone programs help overcome a few important obstacles in treatment, such as engaging patients who might not be reached by traditional in-person treatment, eliminating travel and waiting time, and allowing more flexible scheduling [25].

Each article selected for analysis met the VR criterion as a VR environment (i.e., using three-dimensional graphics presented with a head-mounted display). Two papers described the use of the Oculus Rift HMD [6,20], and one paper [21] only mentioned that VR goggles were used (it is not known what brand). Additionally, each article was based on innovative training designed for specific research. As they are a non-standard therapy, it is worth explaining their structure.

The first article [20] is based on a VR-ICT. One of the goals of a VR-ICT is to maximize the generalization of inhibitory responses to a real-world experiment, where a participant is likely to experience urges to binge eat. Researchers designed a training area without additional distraction, where participants can use hand controllers to control virtual objects. The VR-ICT was based on a GNG (go/no-go) task, in which participants were presented with 3 blocks of 10 trails of stimuli, with 33% of stimuli being binge food (such as pizza, brownies, cake), 33% being fruits and vegetables, and 33% being neutral items (such as bowls, knives, spoons). Results from this initial pilot indicated that delivering ICT through VR was feasible, acceptable, and associated with reductions in binge eating.

The next article [6] was on a MUVR remote VR system, which is a new VR technology with the prospect of supporting remote therapy sessions. Participants and therapists are represented in a virtual environment as cartoon avatars. The overall approach to intervention for body shape and weight concerns utilized to create the content of MUVR remote psychotherapy was based on ACT (acceptance and commitment therapy). For the purposes of this project, the component of values was chosen as the motivational force within ACT, which provides meaning for behavior change to occur, even in the presence of uncomfortable thoughts and feelings. Two virtual tasks were developed within the MUVR system, where the individual comes into contact with valued life domains and recognizes how actions related to concerns about body shape and weight get in the way of living a valued life. The therapist within the MUVR system, located in the same VE as the user, guides the participant to explore what is important to the person. In comparison to conventional VR psychotherapy, MUVR may have the advantage of providing the patients with an anonymous medium to communicate their inner thoughts and feelings, limiting social stigma. The study presents the development of a MUVR program to be utilized as an intervention medium for females at high risk for developing an ED. It also examines the feasibility and acceptability of using VR for remote psychotherapy by therapists and participants at high risk for developing an ED. MUVR may present the advantage of providing patients with an anonymous non-threatening and non-judgmental platform to communicate thoughts and feelings without removing the availability of a real therapist.

In the third article [21], patients were exposed to physical activity simulated in a VR environment. Patients wore VR goggles and watched a jogger running from the first-person perspective as if it were them actually jogging. The jogger’s running video was created using a 360 camera. Patients are able to turn their heads to both sides while jogging and to look up to the sky or down at their shoes. It was also possible to turn their heads back and take a look at the surroundings behind them. They were instructed to devote themselves to the running experience and to engage in simple movements if it facilitated the experience. Patients were asked to rate their acute urge to be physically active during the exposure procedure. The conclusion was that VR could be added to the general treatment of eating disorders as an additional treatment component addressing the acute urge to be physically active would lead to additional benefits for patients who suffer from them.

### 3.3. Outcomes

Given the good results that were achieved by virtual-reality-based therapy in terms of eating disorders, future studies might want to explore the potential of alternative interventions. However, at present, the interest in virtual-reality-based therapy does not appear to have waned yet, as shown by the number of recent studies that have attempted to obtain results by combining virtual-reality-based therapy with other therapies, such as CBT [5,9,10,11]. It is possible that combining virtual-reality-based therapy with other interventions will result in a more successful intervention, as multiple aspects of purging addiction are individual and dependent on the disease entity. Additionally, interventions that used some form of body intervention, such as full body illusions or avatars, have shown promising results as a VR body exposure therapy [7,18,19,22,23,24,25]. More research work will be needed to solidify these findings, especially as some of the studies reported were merely intended as clinical studies for one person [25].

## 4. Discussion

### 4.1. About the Findings

The analysis was based on data from 319 papers; only 15 papers were selected. Our review demonstrates that VR-based therapies are effective in provoking realistic reactions to stimuli environments—a usual treatment can sometimes be even better in the non-immersive world. There are several options for treating EDs, although the review shows that there are many possible combinations and new alternatives. The reviewed papers show their potential utility in reducing, for example, binge eating and increasing justification for change, improving self-esteem, and correcting body view disturbances. There are different ways to use VR to treat EDs because VR can offer an alternative or an addition to interventions. A great innovation is definitely the use of a 360 camera to record a patient’s own intervention video, which can later be displayed using VR goggles. This can allow patients to create an experience where the environment can be adapted, which can further enhance the effectiveness of interventions. VR-exposure therapy can be used to render real-life eating situations in a more life-like and convincing way than traditional methods. Thanks to its design, VR allows for the immersive involvement of not only respondents but also researchers, as both of them can participate in the VR session simultaneously. The possibility of using VR at home is also a big advantage, which makes it easier to conduct sessions. Most of the mentioned VR devices that have been used for research are commercially available to everyone, which increases their usability by researchers, clinicians, and patients. The VR section is also improved by eye-tracking, 360 cameras, and remote therapy. Therefore, it can be concluded that VR technology is getting closer to everyday use and is becoming an indispensable tool now and not just in a distant, inaccessible future. Many of the software used by the researchers are available online, which also increases their availability

### 4.2. Limitations

The first limitation is related to the sample sizes and sample populations reported in the studies. Sample sizes varied extensively (between 1 and 193). Bigger sample sizes increase the chance of finding small effects and reduce the chance of false negatives.. In addition to the size of the sample, not all researchers added a control group between the VR treatment and TAU or between patients and healthy people. Only a few studies included control groups or a follow-up measure.

The second limitation concerns the VR equipment. Some articles did not list the names of the brand of VR equipment (hardware) and software that were used. Only generic names, such as monitor, glasses, or VR goggles, were mentioned.

Much research has focused on different EDs, so the comparison of the papers had to be done separately in three different subsections to allow a seamless comparison.

## 5. Conclusions

The studies presented in this review suggest that VR-based exposure therapy in EDs can be considered a promising addition to treatment as the usual therapy or as a self-sufficient therapy. Moreover, the potential of VR in combination with different therapies may offer an alternative for future research. More rigorous testing, especially in terms of larger sample sizes, the inclusion of control groups or multisessions, and follow-up measures, is still needed. Virtual environments that promote positive stimuli combined with eating disorder knowledge could prove to be a valuable tool for patients with AN, BN, BD, BED, BID, and so forth. The current state of research highlights the importance of the nature and content of VR interventions for ED patients. Future research should look to incorporate more home-based forms of VR tools. Searching for studies based on HMD 3DVR exposure was laborious, and this proves the lack of studies on this subject.

## Figures and Tables

**Table 1:** The short version of the overview table in Supplementary S2, with details of all publications selected for this review.

Reference	Diagnosis *	Methods * (Questionnaires, Protocols, Scales, Measures)	Methods (Hardware and Software)
Porras-Garcia et al. (2019) [22] ^2^	BD	EDI-3	HTC-VIVE eye-tracking (Pupil Labs)
	BID	EDI-BD VR-exposure	Blender 2.78 [26] Unity 3D 5.5 [27]
Nameth et al. (2021) [10] ^1^	BN	VR-CET	Oculus Rift Oculus sensors MSI laptop
	BED	VAS	Unity 3D 5.5.3 [27]
Purvis et al. (2015) [24] ^2^	BD	EDE-Q	VR-HMD NVisor SX111 2560 × 1024
	BID	BMI	Intersense3Cube (accelerometer)
		WSC	Worldviz PPT-H (camera system)
		VR-exposure	Worldviz’s Vizard VR toolkit [28]
Pla-Sanjuanelo et al. (2017/2019) [11] ^1^	BN	EDI-3	IntelPentumT4400 IV 4 GB (laptop)
	BED	EDI-B	15.6-inch stereoscopic monitor
		BMI VR-CET	Earphones Polarized glasses VR-based software for CET
Ferrer-Garcia et al. (2018) [9] ^1^	BN BED	VR-CET A-CBT	15.6-inch 3D laptop Earphones Polarized glasses VR-based software for CET
Manasse et al. (2021) [20] ^3^	LOC BED	EDE TRAQ TEQ UPPS DERS Sham Taste Test VR-ICT	Acer Predator Helios 300 laptop, Oculus Rift Hand controllers and sensors Maya 3D 2016.5 [29]
Porras-Garcia et al. (2021) [8] ^2^	AN	VAS BMI, PASTAS EDI-3 BAS BIAS-BD TAU VR-exposure	HTC-VIVE HMD FOVE VR-HMD (for eye detectors) Blender 2.78 [26] Unity 3D 5.6.1 [27]
Matsangidou et al. (2020) [6] ^3^	AN (or high risk) BN (or high risk)	WSC EDDS MUVR-exposure	Oculus Rift HMD Oculus Touch Controller Oculus Sensors Unity3D 5.0 [27] Steam VR [30] Adobe Fuse CC [31] Maya 2016.5 [29] Salsa Lip-Sync RandomEyes 1.5.5 [32] PhotonVoice 1.16 [33] Photon Unity Networking 1.91 [34] Open Broadcaster 23.0.1 [35]
Porras-Garcia et al. (2020) [23] ^2^	AN BD	BMI EDI-3 EDI-BD PASTAS BIAS-BD BAS VAS VR-exposure	HTC-VIVE HMD Computer with Nvidia RTX 2080 (graphic card) HMD FOVE (Eye-Tracking) Hand controllers Body trackers FOVE setup 0.16.0 Unity 3D 3.0.0 [27] Blender 2.78 [26]
Mountford et al. (2015) [7] ^2^	BD BID	EDE-Q Restraint Scale FNAES LSAS BISS VAS VR-exposure	nVisor SX111HMD Headphones Unity software package by Ari Jacobs of ‘‘I’m VR’’ IS900 VET tracking system
Serino et al. (2019) [25] ^2^	AN	Embodiment Questionnaire, VR-exposure	Oculus Rift DK2 Razer Hydra MakeHuman [36] Unity3D 3.0 [27]
Irvine et al. (2020) [18] ^2^	BD BID	EDE-Q BSQ-16b BDI RSE BMI VR-exposure	Oculus Rift with Touch controllers Unreal Engine 4.18 [37]
Keizer et al. (2016) [19] ^2^	AN	BAT EDI-II VR-exposure, EQ	Oculus Rift DK2 Razer Hydra Unity3D 2.5.5 [27] Blender 2.1 [26] MakeHuman [36]
Paslakis et al. (2017) [21] ^3^	AN BN	BMI SmQ EDE-Q EDS-21 CET-VR saliva samples (a-amylase, cortisol, and cortisone) and blood (leptin)	360 camera fixed on the helmet VR goggles
Ferrer-Garcia et al. (2017) [5] ^1^	BN BED	EDI-3 FCQ-T STAI-S STAI-Y STAI-T FCQ-S FCQ-T VR-CET	15.6-inch sterescopic monitor Earphones Polarized glasses VR-CET software

* Abbreviations are explained in Supplementary S2. ^1^ VR-CET intervention; ^2^ VR-exposure; ^3^ alternative form.

## Data Availability

Not applicable.

## Supplementary Materials

The following supporting information can be downloaded at: https://www.mdpi.com/article/10.3390/jcm11174956/s1, Supplementary S1: PRISMA checklist. Supplementary S2: Overview table with details of all publications selected for this review, including details about the study objectives, design, diagnostics, sample size, control group (if any), session procedures, methods, intervention (VR-task), and outcomes.
